# Comprehensive genomic sequencing detects important genetic differences between right-sided and left-sided colorectal cancer

**DOI:** 10.18632/oncotarget.20510

**Published:** 2017-08-24

**Authors:** Yoshifumi Shimada, Hitoshi Kameyama, Masayuki Nagahashi, Hiroshi Ichikawa, Yusuke Muneoka, Ryoma Yagi, Yosuke Tajima, Takuma Okamura, Masato Nakano, Jun Sakata, Takashi Kobayashi, Hitoshi Nogami, Satoshi Maruyama, Yasumasa Takii, Tetsu Hayashida, Hiromasa Takaishi, Yuko Kitagawa, Eiji Oki, Tsuyoshi Konishi, Fumio Ishida, Shin-ei Kudo, Jennifer E. Ring, Alexei Protopopov, Stephen Lyle, Yiwei Ling, Shujiro Okuda, Takashi Ishikawa, Kohei Akazawa, Kazuaki Takabe, Toshifumi Wakai

**Affiliations:** ^1^ Division of Digestive and General Surgery, Niigata University Graduate School of Medical and Dental Sciences, Niigata, Japan; ^2^ Department of Surgery, Niigata Cancer Center Hospital, Niigata, Japan; ^3^ Department of Surgery, Keio University School of Medicine, Tokyo, Japan; ^4^ Department of Internal Medicine, Keio University School of Medicine, Tokyo, Japan; ^5^ Department of Surgery and Science, Graduate School of Medical Sciences, Kyushu University, Fukuoka, Japan; ^6^ Department of Gastroenterological Surgery, Gastroenterological Center, Cancer Institute Hospital, Japanese Foundation for Cancer Research, Tokyo, Japan; ^7^ Digestive Disease Center, Showa University Northern Yokohama Hospital, Yokohama, Japan; ^8^ KEW, Inc., Cambridge, MA, USA; ^9^ University of Massachusetts Medical School, Worcester, MA, USA; ^10^ Division of Bioinformatics, Niigata University Graduate School of Medical and Dental Sciences, Niigata, Japan; ^11^ Department of Medical Informatics, Niigata University Graduate School of Medical and Dental Sciences, Niigata, Japan; ^12^ Breast Surgery, Roswell Park Cancer Institute, Buffalo, NY, USA; ^13^ Department of Surgery, University at Buffalo Jacobs School of Medicine and Biomedical Sciences, The State University of New York, Buffalo, NY, USA

**Keywords:** colorectal cancer, right-sided, anti-EGFR therapy, next-generation sequencing, comprehensive genomic sequencing

## Abstract

**Objectives:**

Anti-epidermal growth factor receptor (EGFR) therapy has been found to be more effective against left-sided colorectal cancer (LCRC) than right-sided colorectal cancer (RCRC). We hypothesized that RCRC is more likely to harbor genetic alterations associated with resistance to anti-EGFR therapy and tested this using comprehensive genomic sequencing.

**Materials and methods:**

A total of 201 patients with either primary RCRC or LCRC were analyzed. We investigated tumors for genetic alterations using a 415-gene panel, which included alterations associated with resistance to anti-EGFR therapy: TK receptors (*ERBB2*, *MET*, *EGFR*, *FGFR1*, and *PDGFRA*), RAS pathway (*KRAS*, *NRAS*, *HRAS*, *BRAF*, and *MAPK2K1*), and PI3K pathway (*PTEN* and *PIK3CA*). Patients whose tumors had no alterations in these 12 genes, theoretically considered to respond to anti-EGFR therapy, were defined as “all wild-type”, while remaining patients were defined as “mutant-type”.

**Results:**

Fifty-six patients (28%) and 145 patients (72%) had RCRC and LCRC, respectively. Regarding genetic alterations associated with anti-EGFR therapy, only 6 of 56 patients (11%) with RCRC were “all wild-type” compared with 41 of 145 patients (28%) with LCRC (*P* = 0.009). Among the 49 patients who received anti-EGFR therapy, RCRC showed significantly worse progression-free survival (PFS) than LCRC (*P* = 0.022), and “mutant-type” RCRC showed significantly worse PFS compared with “all wild-type” LCRC (*P* = 0.004).

**Conclusions:**

RCRC is more likely to harbor genetic alterations associated with resistance to anti-EGFR therapy compared with LCRC. Furthermore, our data shows primary tumor sidedness is a surrogate for the non-random distribution of genetic alterations in CRC.

## INTRODUCTION

The colon is an embryological derivative of the midgut and hindgut separately, and thus the right-sided colon, the left-sided colon, and the rectum each have different anatomical and physiological features. Evidence shows that tumors arising from right colon have distinct clinical and biological characteristics compared with tumors of the left colon or rectum [1-6]. Right-sided colorectal cancer (RCRC) is generally characterized by being more common in women, and associated with Lynch syndrome, the serrated pathway, Mitogen-activated protein kinase signaling, microsatellite instability-high (MSI-H), deficiency of mismatch repair genes, CpG island methylation, and *KRAS* and *BRAF* mutations [7-10]. Left-sided colorectal cancer (LCRC) is more common in men, and associated with familial adenomatous polyposis syndrome, Wnt and EGFR signaling, chromosomal instability, *ERBB1* and *ERBB2* amplifications, and *APC*, *p53*, and *NRAS* mutations [10, 11]. Based on these molecular differences, sidedness of CRC is thought to be associated with efficacy of chemotherapy and targeted therapy.

The monoclonal antibodies cetuximab and panitumumab are epidermal growth factor receptor (EGFR) inhibitors that block downstream signaling of the EGFR pathway. Randomized phase III clinical trials have shown a survival benefit of these anti-EGFR monoclonal antibodies in *RAS* wild-type metastatic CRC [12-14]; however, tumor location has not traditionally been included as a stratification criterion in clinical trials. Recently, several retrospective, unplanned analyses examined primary tumor sidedness and revealed that anti-EGFR therapy clearly benefitted patients with LCRC, whereas patients with RCRC derived limited benefit [15-17]. Consequently, while these analyses were limited by low numbers of RCRC patients, the related imbalance between groups, and no randomization; primary tumor sidedness of CRC has emerged as new predictive marker for efficacy of anti-EGFR therapy.

The mechanism of resistance to anti-EGFR therapy in patients with RCRC has not been fully elucidated. Although *RAS* mutations are established biomarkers of efficacy to anti-EGFR therapy, anti-EGFR therapy is not effective for all patients with a *RAS* wild-type tumor [18-21]. Genetic alterations in tyrosine kinase (TK) receptors, the RAS pathway (other than *KRAS* and *NRAS* mutations), and the PI3K pathway are other possible mechanisms of resistance to anti-EGFR therapy [22, 23]. While the most clinically important alterations, such as *KRAS*, *NRAS*, and *BRAF* mutations, have been widely analyzed among patients with metastatic CRC, the other alterations have not been widely studied.

Next-generation sequencing projects, such as The Cancer Genome Atlas, have profiled genomic changes in many cancers including CRC [24]. We have similarly reported a genomic analysis of Japanese CRC patients using comprehensive genomic sequencing (CGS) [25, 26]. CGS detects gene mutations and copy number alterations in TK receptors, and the RAS and PI3K pathway in a single assay. In the present analysis, we hypothesized that RCRC more frequently harbors genetic alterations associated with resistance to anti-EGFR therapy compared with LCRC. To test this hypothesis, we investigated these genetic alterations using CGS.

## RESULTS

### Association between primary tumor sidedness and clinicopathological characteristics

**Figure 1:**
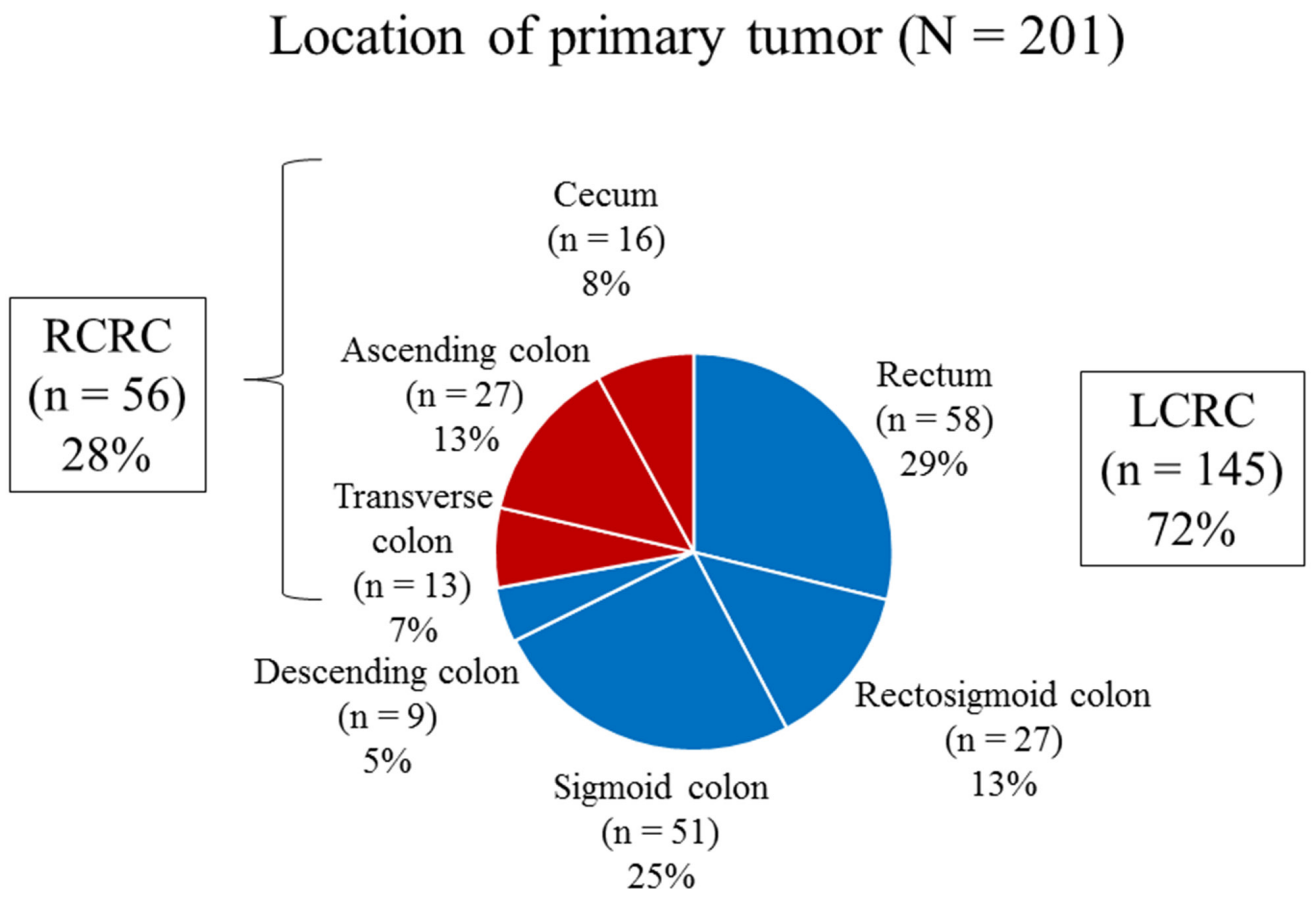
Primary tumor locations in right-sided colorectal cancer and left-sided colorectal cancer RCRC, right-sided colorectal cancer; LCRC, left-sided colorectal cancer.

Fifty-six patients (28%) and 145 patients (72%) had RCRC and LCRC, respectively (Figure 1). Histopathological grade 3 was significantly associated with RCRC (*P* < 0.001; Table 1). Medullary type, mucinous type, and MLH1/MSH2 status were significantly associated with RCRC (*P* = 0.022, *P* = 0.007, and *P* = 0.024, respectively; Table 2).

**Table 1:** Association between primary tumor sidedness and clinicopathological characteristics (N = 201)

	Primary tumor sidedness		*P*-value
Variable	Right (n = 56)	Left (n = 145)	
Age (years)			
< 65	22	78	0.065
≥ 65	34	67	
Sex			
Male	29	55	0.074
Female	27	90	
Tumor size (mm)			
< 50	23	65	0.630
≥ 50	33	80	
T category			
T1, 2	6	18	0.739
T3, 4	50	127	
Histopathological grading			
G1, 2	31	116	< 0.001
G3	25	29	
Lymphatic invasion			
Absence	18	61	0.196
Presence	38	84	
Venous invasion			
Absence	10	38	0.213
Presence	46	107	
N category			
N0	13	46	0.235
N1, 2	43	99	
M category			
M0	22	68	0.331
M1	34	77	

**Table 2:** Primary tumor sidedness and pathological and genetic characteristics related with deficiency of mismatch repair genes (N = 201)

	Primary tumor sidedness		*P*-value
Variable	Right (n = 56)	Left (n = 145)	
Medullary type			
Yes	52	144	0.022
No	4	1	
Mucinous type			
Yes	47	139	0.007
No	9	6	
Signet ring type			
Yes	55	143	0.999
No	1	2	
Tumor infiltrating lymphocytes^a^			
Yes	13	23	0.223
No	43	122	
MLH1/MSH2 status			
Normal	22	69	0.013
Abnormal	10	9	
Hypermutated phenotype			
Hypermutated	10	7	0.008
Non-hypermutated	46	138	

^a^ Cut-off value = 10 lymphocytes/5 high power fields.

### Association between primary tumor sidedness and genetic alterations evaluated using CGS

**Figure 2:**
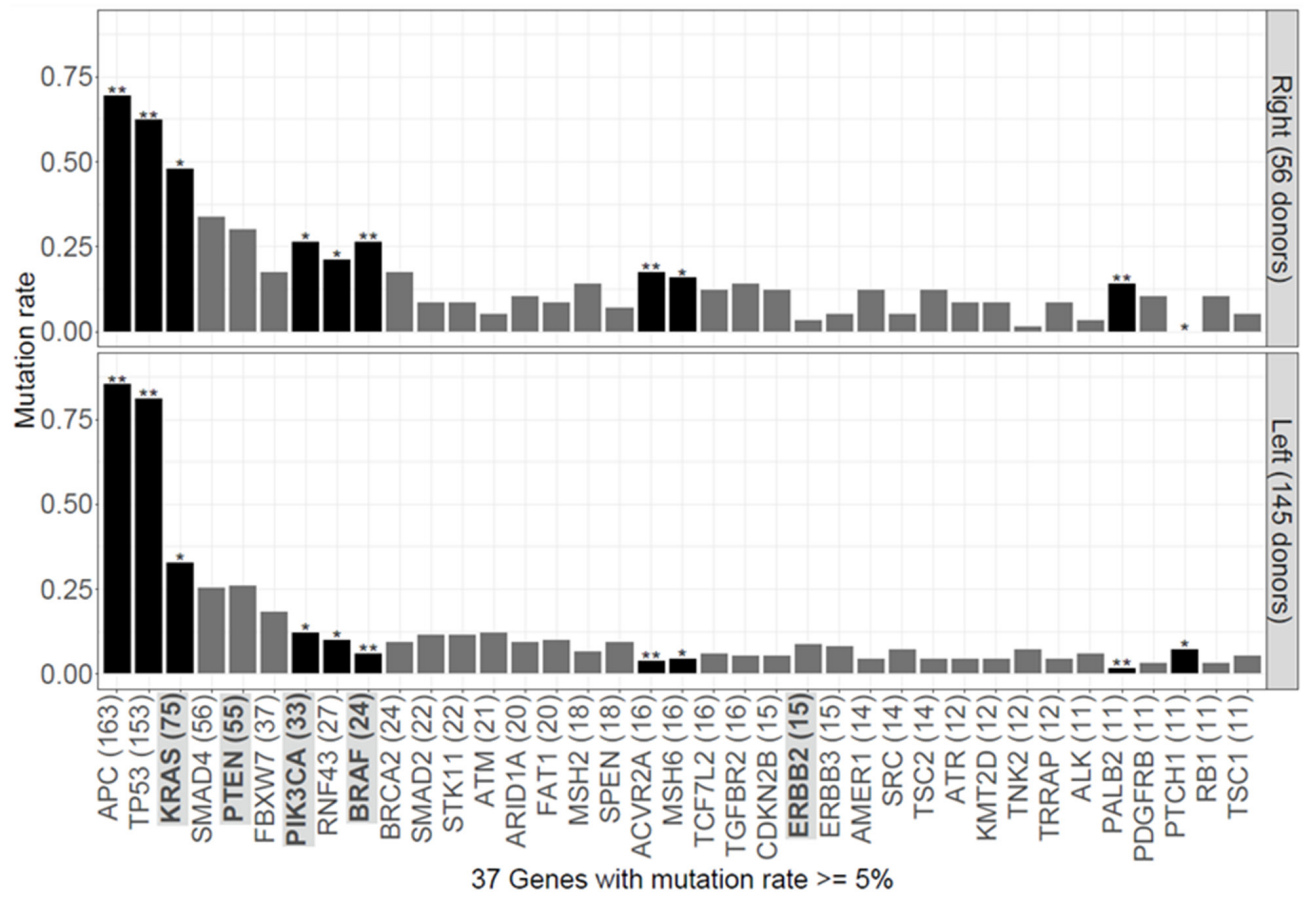
Distribution of genetic alterations in right-sided and left-sided colorectal cancer Dark bars indicate genes with a significant difference (*P* < 0.05, two-tailed Fisher’s exact test or Chi-squared test) in the frequency of genetic alterations compared with other-sided donors. Light bars indicate genes that are not significantly different (^*^, *P* < 0.05; ^**^, *P* < 0.01). The genes associated with anti-EGFR resistance were highlighted.

**Figure 3:**
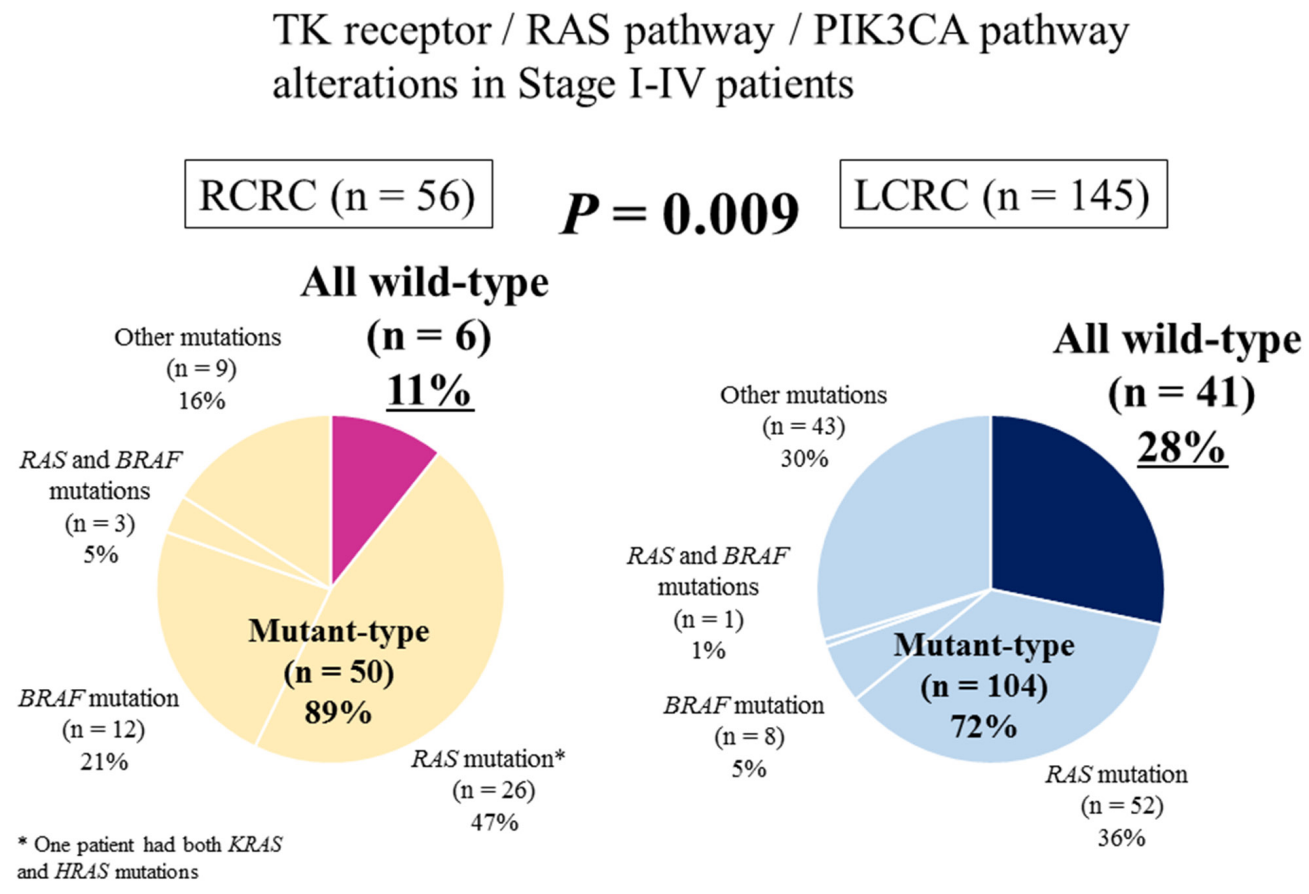
Percentage of genetic alterations associated with resistance to anti-EGFR therapy in right-sided colorectal cancer and left-sided colorectal cancer Genetic alterations in TK receptors (*ERBB2, MET, EGFR, FGFR1*, and *PDGFRA*), RAS pathway (*KRAS, NRAS, HRAS, BRAF*, and *MAPK2K1*), and PI3K pathway (*PTEN* and *PIK3CA*) were evaluated using comprehensive genomic sequencing of the 415-gene panel. Patients who had no alterations in all 12 genes were defined as “all wild-type”. RCRC, right-sided colorectal cancer; LCRC, left-sided colorectal cancer.

CGS of the 415-gene panel in our cohort of 201 patients detected genetic alterations in 268 genes (Supplementary Table 1). Mutations in *KRAS*, *PIK3CA*, *RNF43*, *BRAF*, *ACVR2A*, *MSH6*, and *PALB2* were significantly associated with RCRC (*P* = 0.047, *P* = 0.014, *P* = 0.039, *P* < 0.001, *P* = 0.003, *P* = 0.016, and *P* = 0.001, respectively; Figure 2), and mutations in *APC*, *TP53* and *PTCH1* were significantly associated with LCRC (*P* = 0.010, *P* = 0.005, and *P* = 0.036, respectively; Figure 2). The hypermutated tumor was significantly associated with RCRC (*P* = 0.008; Table 2). Genetic alterations found in the 12 genes associated with resistance to anti-EGFR therapy (TK receptors: *ERBB2*, *MET*, *EGFR*, *FGFR1*, and *PDGFRA*; RAS pathway: *KRAS*, *NRAS*, *HRAS*, *BRAF*, and *MAPK2K1*; and PI3K pathway: *PTEN* and *PIK3CA*) are detailed in Supplementary Table 2. Of the 201 patients, 154 harbored one or more genetic alterations in these 12 genes with 80, 63, 8, 2, and 1 patients had 1, 2, 3, 4, and 5 gene alterations, respectively. Of the 56 RCRC patients, 6 (11%) were wild-type in all 12 genes (termed “all wild-type”); while 41 of 145 patients (28%) of LCRC were “all wild-type” (*P* = 0.009; Figure 3, Table 3).

**Table 3:** Association between primary tumor sidedness and gene alterations of TK receptors/RAS pathway/PI3K pathway (N = 201)

	Primary tumor sidedness		*P*-value
Variable	Right (n = 56)	Left (n = 145)	
*ERBB2* status			
Wild-type	54	132	0.243
Mutant^a^	2	13	
*MET* status			
Wild-type	55	139	0.676
Mutant^a^	1	6	
*EGFR* status			
Wild-type	56	141	0.578
Mutant	0	4	
*FGFR1* status			
Wild-type	56	135	0.065
Mutant	0	10	
*PDGFRA* status			
Wild-type	55	144	0.481
Mutant ^a^	1	1	
*KRAS* status			
Wild-type	29	97	0.047
Mutant	27	48	
*NRAS* status			
Wild-type	54	142	0.620
Mutant	2	3	
*HRAS* status			
Wild-type	55	143	0.999
Mutant	1	2	
*BRAF* status			
Wild-type	41	136	< 0.001
Mutant	15	9	
*MAPK2K1* status			
Wild-type	54	137	0.729
Mutant	2	8	
*PTEN* status			
Wild-type	39	107	0.554
Mutant ^b^	17	38	
*PIK3CA* status			
Wild-type	41	127	0.014
Mutant	15	18	
Alterations in TK receptors/RAS pathway/PI3K pathway			
0	6	41	0.024
1	24	56	
2 or more	26	48	
All wild-type	6	41	0.009
Mutant-type	50	104	

^a^ Including mutation and amplification.

^b^ Including mutation and deletion

### Efficacy of anti-EGFR therapy according to primary tumor sidedness and genetic alterations associated with resistance to anti-EGFR therapy

**Figure 4:**
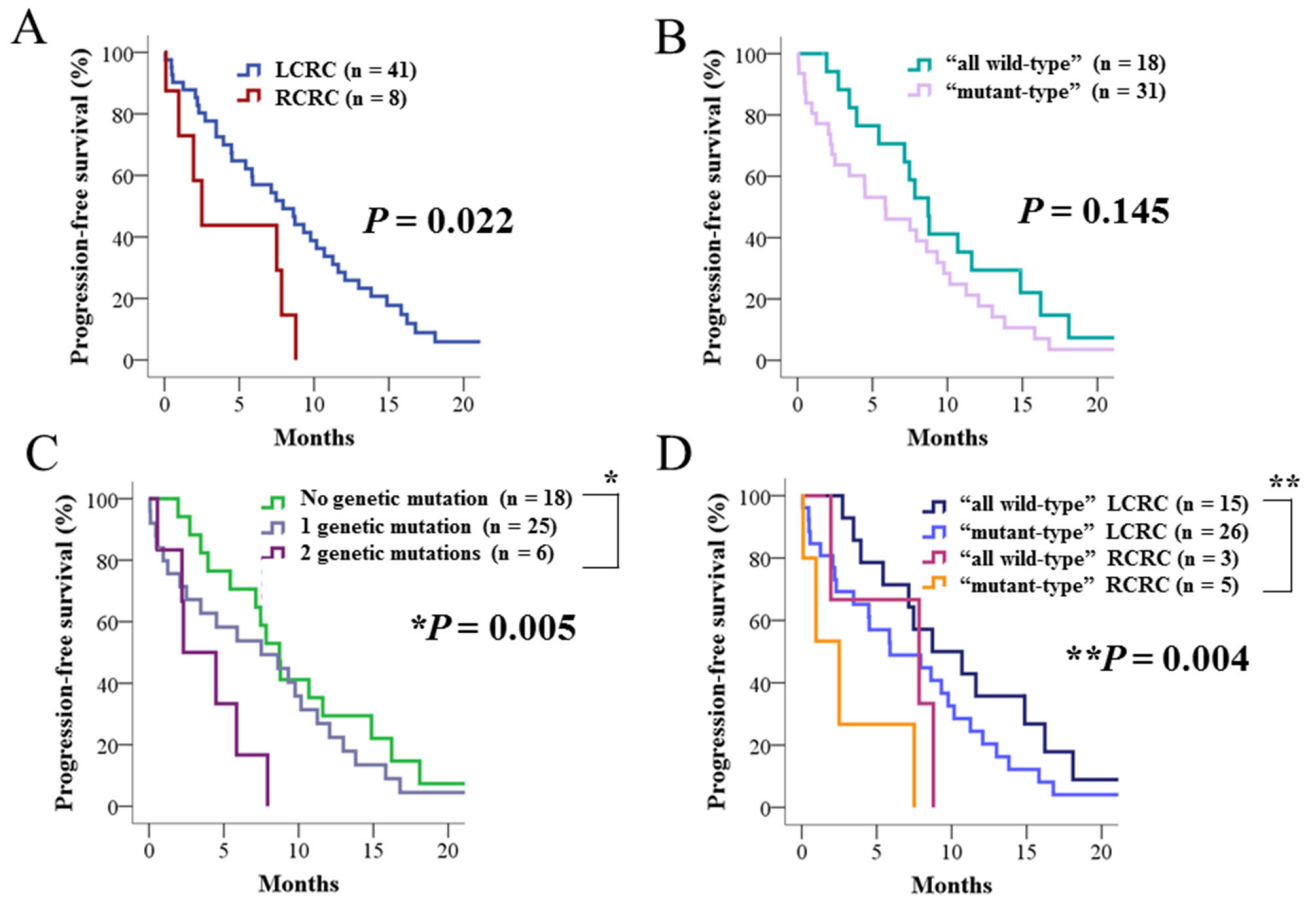
Progression-free survival of patients who received anti-EGFR therapy in addition to cytotoxic chemotherapy **(A)** Progression-free survival stratified by primary tumor sidedness. **(B)** Progression-free survival stratified by genetic alterations associated with resistance to anti-EGFR therapy. **(C)** Progression-free survival stratified by the number of genetic alterations associated with resistance to anti-EGFR therapy. **(D)** Progression-free survival stratified by primary tumor sidedness and genetic alterations. “All wild-type” indicates patients without any genetic alterations associated with resistance to anti-EGFR therapy, and “mutant-type” indicates those with one or more genetic alterations. RCRC, right-sided colorectal cancer; LCRC, left-sided colorectal cancer.

Among the 49 patients treated with anti-EGFR therapy in addition to cytotoxic chemotherapy, patients with RCRC showed significantly worse progression-free survival (PFS) than patients with LCRC (*P* = 0.022; Figure 4A). Regarding the 12 genes associated with anti-EGFR therapy resistance in these 49 patients, 18, 25, and 6 patients had 0, 1 and 2 genetic alterations, respectively. No significant difference was observed in PFS between “all wild-type” and “mutant-type” (Figure 4B), but the six patients with two genetic alterations showed significantly worse PFS than patients with no genetic mutations (*P* = 0.005; Figure 4C). After stratification by primary tumor sidedness, we found *BRAF* mutations were significantly associated with RCRC (*P* = 0.047; Table 4). When the 49 patients were classified into four groups according to primary tumor sidedness and genetic alterations associated with anti-EGFR therapy resistance, “mutant-type” RCRC showed a significantly worse PFS compared with “all wild-type” LCRC (*P* = 0.004; Figure 4D).

**Table 4:** Association between primary tumor sidedness and clinicopathological characteristics in 49 patients with anti-EGFR therapy in addition to cytotoxic chemotherapy

	Primary tumor sidedness		*P*-value
Variable	Right (n = 8)	Left (n = 41)	
Age (years)			
< 65	4	27	0.443
≥ 65	4	14	
Sex			
Male	4	27	0.443
Female	4	14	
Tumor size (mm)			
< 50	5	19	0.463
≥ 50	3	22	
T category			
T2, 3	4	18	0.999
T4	4	23	
Histopathological grading			
G1, 2	3	32	0.033
G3	5	9	
Lymphatic invasion			
Absence	1	14	0.406
Presence	7	27	
Venous invasion			
Absence	2	6	0.601
Presence	6	35	
N category			
N0	1	5	0.999
N1, 2	7	36	
M category			
M0	1	1	0.421
M1a	4	23	
M1b	3	17	
*KRAS* status			
Wild-type	7	39	0.421
Mutant	1	2	
*BRAF* status			
Wild-type	5	38	0.047
Mutant	3	3	
Alterations in TK receptors/RAS pathway/PI3K pathway excluding *KRAS* and *BRAF* mutations			
Absence	7	20	0.059
Presence	1	21	
Alterations in TK receptors/RAS pathway/PI3K pathway			
0	3	15	0.492
1	5	20	
2	0	6	
All wild-type	3	15	0.999
Mutant-type	5	26	
Anti-EGFR drug			
Cetuximab	2	9	0.999
Panitumumab	6	32	
Anti-EGFR therapy			
Initial therapy	3	9	0.386
Subsequent therapy	5	32	
Chemotherapy added to anti-EGFR therapy			
Oxaliplatin-based	3	7	0.195
Irinotecan-based	3	29	
Anti-EGFR drug only	2	5	

## DISCUSSION

CGS analysis of genetic alterations in 201 primary CRCs revealed important genetic differences in relation to tumor sidedness: that there are genetic alterations in RCRC that are distinct from LCRC, and that CRCs wild-type in TK receptors and the RAS and PI3K pathways (termed “all wild-type” tumors and theoretically more likely to respond to anti-EGFR therapy), were significantly less common amongst RCRC. These genetic differences likely drive the inherent resistance of RCRCs to anti-EGFR therapy.

Primary tumor sidedness of CRC has prognostic importance and relates to response to targeted therapy [15-18]. Recent meta-analyses reported that RCRC was a negative prognostic variable independent of Tumor-Node-Metastasis (TNM) stage [16]. Furthermore, patients with *RAS* wild-type LCRC had significantly greater survival benefit from anti-EGFR therapy compared with anti-vascular endothelial growth factor (VEGF) therapy; and, conversely, RCRC had poor benefit from standard therapies including anti-EGFR therapy, but was associated with longer survival with anti-VEGF therapy [17, 18]. The National Comprehensive Cancer Network (NCCN) guidelines noted that cetuximab and panitumumab confer little, if any, benefit to patients with metastatic CRC if the primary tumor originated on the right side, and primary tumor sidedness is a surrogate for the non-random distribution of molecular subtypes [18]. However, the molecular background of RCRC and LCRC has not been fully elucidated, and it is still unclear why anti-EGFR therapy is less efficacious in patients with RCRC compared with patients with LCRC. Hence, we investigated genomic differences between RCRC and LCRC using CGS, focusing on identifying the mechanism driving the observed difference in response to anti-EGFR therapy.

CGS has been shown to detect numerous genetic alterations, including driver mutations, in many solid cancers [24, 25]. Mutations in the RAS pathway, such as *KRAS*, *NRAS*, and *BRAF*, are benchmarks to determine treatment strategies for patients with metastatic CRC. The NCCN guidelines state that all patients with metastatic CRC should have tumor tissue genotyped for *KRAS*, *NRAS*, and *BRAF* mutations, and patients with any known *KRAS* or *NRAS* mutation should not be treated with anti-EGFR therapy such as cetuximab and panitumumab [18]. In the present analysis, we assumed that genetic alterations in TK receptors, the RAS pathway, or the PI3K pathway are possible mechanisms underlying resistance to anti-EGFR therapy [22, 23]. We successfully detected genetic alterations, not only in the RAS pathway, but also among TK receptors and the PI3K pathway that may be associated with resistance to anti-EGFR therapy. Furthermore, patients with RCRC showed a significantly worse PFS than those with LCRC.

Cancer genome profiling seeks to enable precision medicine, modifying therapies based on the unique genomic changes inherent in the individual tumor of each patient. In the present analysis, we showed an association between tumor sidedness and gene mutations, which may explain the difference in efficacy of anti-EGFR therapy in RCRC compared with LCRC. The genomic background of RCRC as revealed by CGS is consistent with the results of previous meta-analyses [15-17] and the NCCN guidelines regarding the relevance of tumor sidedness [18]. However, we also demonstrated that approximately 10% of RCRC patients had the “all wild-type” phenotype with no mutations detected in TK receptors or the RAS or PI3K pathways, and therefore, theoretically, these patients would be considered as responders to anti-EGFR therapy despite having RCRC. As such, while we showed that RCRC commonly demonstrates a genomic profile associated with resistance to anti-EGFR therapy, we propose future analyses should focus on individual tumors rather than primary tumor sidedness to best facilitate precision medicine.

CGS has ability to detect numerous actionable mutations that can guide new treatment strategies. In this analysis, a novel finding was *PALB2* mutations occurring significantly more frequent in RCRC than LCRC. *PALB2* is a DNA maintenance gene, where the encoded protein binds to and colocalizes with BRCA2 in nuclear foci, and plays a role of tumor suppression [27]. In CRC, the significance of *PALB2* mutations has not been elucidated, and this is the first report regarding *PALB2* mutations in relation to CRC sidedness. *PALB2* mutations are considered to be actionable, and are a biomarker for response to Poly (ADP-ribose) polymerase (PARP) inhibitors in pancreatic (ClinicalTrials.gov Identifier: NCT03140670) and prostatic (ClinicalTrials.gov Identifier: NCT02952534) cancers. In this analysis, we found 8 of 56 (14%) RCRCs had *PALB2* mutations compared with 3 of 145 (2%) LCRCs. Thus, targeting PALB2 may represent a future treatment strategy for RCRC.

To the best of our knowledge, this is the first report describing a genomic overview of RCRC and LCRC using CGS. However, this analysis has several limitations. First, it was a retrospective analysis performed at two institutions and included a relatively small number of patients. Second, the selection of genomic biomarkers of resistance outside of *RAS* is not yet well supported by prospective studies. Third, as patients who received anti-EGFR therapy were analyzed retrospectively, we could not definitively associate primary tumor sidedness with response to anti-EGFR therapy. Fourth, as the number of RCRC patients who received anti-EGFR therapy was small, we need for increasing the number of RCRC patients in future. However, we did demonstrate that RCRC was significantly associated with genetic alterations associated with resistance to anti-EGFR therapy, which provides a plausible mechanism of resistance to anti-EGFR therapy in patients with RCRC.

In conclusion, we show RCRC is more likely to harbor genetic alterations associated with resistance to anti-EGFR therapy compared with LCRC, and primary tumor sidedness is a surrogate for a non-random distribution of genetic alterations in CRC.

## MATERIALS AND METHODS

### Patients

This retrospective analysis was performed in accordance with the Helsinki Declaration and the protocol was approved by the Ethics Committee of the School of Medicine, Niigata University. We randomly selected and enrolled 201 patients diagnosed with stage I - IV CRC based on the 7^th^ edition of the American Joint Committee on Cancer staging manual [28] who had a primary tumor resection between 2009 and 2015 at Niigata University Medical and Dental Hospital or Niigata Cancer Center Hospital. In this analysis, we included the 201 independent individuals, all unrelated, confirmed from our database and medical charts. Patients with familial adenomatous polyposis or inflammatory bowel disease were excluded.

### Primary tumor sidedness and clinicopathological characteristics

Primary tumor location was determined by operative findings. Cancer in the cecum, ascending colon, hepatic flexure, or transverse colon was classified as right-sided; and cancer in the splenic flexure, descending colon, sigmoid colon, rectosigmoid, or rectum was classified as left-sided [29, 30]. Histopathological features associated with RCRC, such as medullary type, mucinous type, signet ring type, and tumor-infiltrating lymphocytes were analyzed by a previously reported method [31]. MutL homologue 1 (MLH1)/MutS homologue 2 (MSH2) status was evaluated in 110 of the 201 patients by immunohistochemistry with anti-MLH1 (1:50; BD Biosciences PharMingen, San Diego, CA) and anti-MSH2 (1:50; Leica Microbiosystems, Tokyo, Japan) antibodies. Hypermutation was defined as a tumor with MSI-H and/or high tumor mutation burden (TMB), as described previously [25], using CGS. TMB was calculated as the number of non-synonymous mutations per megabase of sequence in the panel (panel size = 1.3 Mb). To be classified as hypermutated, the threshold of TMB was set as the lowest TMB observed in tumors with MSI-H. Tumors with mutations in *POLE* or other DNA repair genes can have very high TMB but not show MSI-H [25].

### CGS analysis of primary tumors

Archival tissue in the form of formalin-fixed, paraffin-embedded (FFPE) tumor or unstained tissue sections obtained during primary tumor resection were used for CGS. An independent pathologist evaluated tumor content in each sample using hematoxylin and eosin-stained slides to ensure > 50% tumor content was present. Where applicable, unstained slides were macro-dissected to enrich for tumor content and DNA was extracted using a BioStic FFPE Tissue DNA Isolation Kit (Mo Bio Laboratories, Inc., Carlsbad, CA). All sample preparation, CGS, and analytics were performed in a CLIA/CAP-accredited laboratory (KEW Inc., Cambridge, MA). DNA fragment (50–150 ng) libraries were prepared and enriched for the CancerPlex 415-gene panel (KEW Inc.) [25, 26], a large clinically validated panel of 415 genes enriched for coding regions and selected introns of known cancer-related genes. Sequencing was performed on Illumina MiSeq and NextSeq platforms with an average 500× sequencing depth. Genomic data were then processed through a proprietary bioinformatics platform and knowledgebase to identify multiple classes of genomic abnormalities including single nucleotide substitutions, small insertions/deletions, copy number variations, and translocations. Single nucleotide variant (SNV) and insertion or deletion (indel) calling were only performed in genomic regions intended to be captured by the assay (region of interest). We set a standard threshold of 10% allelic fraction for calling SNVs and indels to focus on primary truncal driver mutations and avoid subclonal events. Copy number variants were called for exons as well as globally. We segmented regions using a Fused-Lasso method and export the results to a VCF file. The threshold for gain was > 2.5 fold and for loss was < 0.5 fold. Variants were filtered or flagged according to technical quality (e.g. coverage, allelic fraction, number of supporting reads), presence in previously characterized normal samples, or presence/absence in the following databases: dbSNP, ExAC, COSMIC, ClinVar, and KEW. SNVs and indels in VCF format were annotated using SnpEff and the output was adapted according to HGVS recommendations [25, 26].

### Genetic alterations in TK receptors and the RAS and PI3K pathways in RCRC and LCRC

Genetic alterations of TK receptors (*ERBB2*, *MET*, *EGFR*, *FGFR1*, and *PDGFRA*), RAS pathway (*KRAS*, *NRAS*, *HRAS*, *BRAF*, and *MAPK2K1*), and PI3K pathway (*PTEN* and *PIK3CA*) were analyzed using CGS of the 415-gene panel. We defined patients who had no alterations in all 12 genes as “all wild-type”; theoretically, these patients should respond to anti-EGFR therapy [22, 23]. We defined the remaining patients with genetic alterations as “mutant-type”. We also estimated the incidence of “all wild-type” for RCRC and LCRC. In this analysis of 201 patients, 49 received anti-EGFR therapy. In these 49 patients, we investigated the efficacy of anti-EGFR therapy according to primary tumor sidedness and genetic alterations associated with resistance to anti-EGFR therapy.

### Statistical analysis

Statistical analyses were performed with IBM SPSS Statistics 22 (IBM Japan, Inc., Tokyo, Japan). A Fisher’s exact test or Chi-squared test was used to evaluate associations between primary tumor sidedness and clinicopathological characteristics, and primary tumor sidedness and genetic alterations were evaluated with CGS. The association between primary tumor sidedness and genetic alterations associated with resistance to anti-EGFR therapy was examined by Fisher’s exact test or Chi-squared test. PFS rates in patients treated with anti-EGFR therapy (cetuximab or panitumumab) in addition to cytotoxic chemotherapy were estimated using Kaplan-Meier analysis. A log-rank test was used to assess for a significant difference between right-sided and left-sided tumors. *P*-values < 0.05 were considered statistically significant.

## SUPPLEMENTARY MATERIAL TABLES






