# Supplementary material for: Comprehensive genomic sequencing detects important genetic differences between right-sided and left-sided colorectal cancer
Source: Oncotarget. 2017 Aug 24;8(55):93567–79. doi: 10.18632/oncotarget.20510 (PMC5706819; doi:10.18632/oncotarget.20510)
Supplement: Supplementary file 1 [file oncotarget-08-93567-s001.pdf]

Comprehensive genomic sequencing detects important genetic differences between right-sided and left-sided colorectal cancer

SUPPLEMENTARY MATERIALS

Supplementary Table 1: Genetic alterations identified in 268 genes in 201 CRC patients.

See Supplementary File 1

Supplementary Table 2: Genetic alterations identified in 12 genes associated with resistance to anti-EGFR therapy in 201 CRC patients.

See Supplementary File 2
